# Supplementary material for: Saccharomyces boulardii (CNCM I-745) improves intestinal damage in sepsis by remodeling intestinal flora structure
Source: Front Cell Infect Microbiol. 2025 Jul 31;15:1602792. doi: 10.3389/fcimb.2025.1602792 (PMC12350329; doi:10.3389/fcimb.2025.1602792)
Supplement: Supplementary file 1 [file DataSheet1.zip › summary/summary/4_Alpha_diversity/5_rank_abundance_graph.pdf]

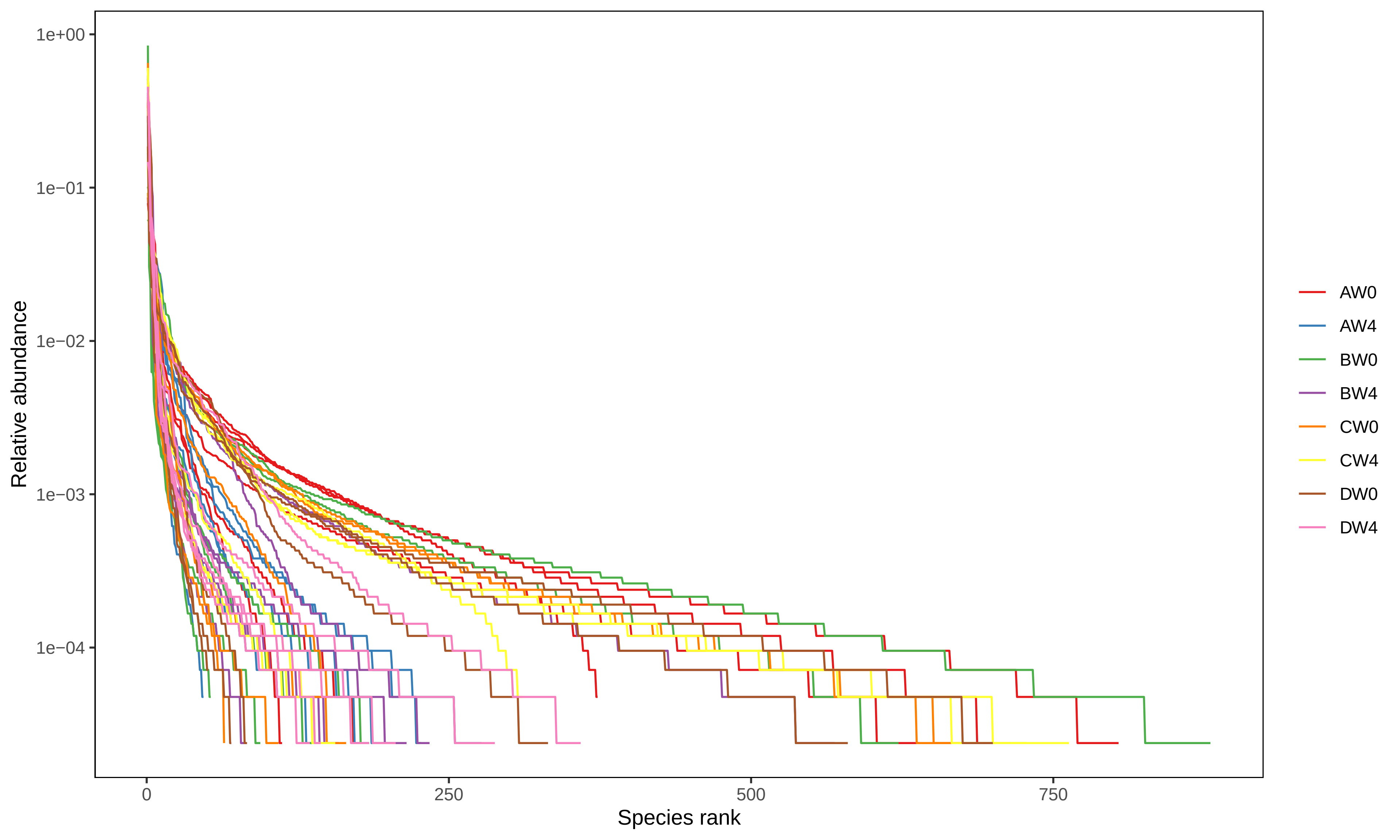
1e+00
1e−01
1e−02
1e−03
1e−04
Relative abundance
0
250
500
750
Species rank
AW0
AW4
BW0
BW4
CW0
CW4
DW0
DW4
